# Relatives of deceased patients with metastatic lung cancer’s views on the achievement of treatment goals and the choice to start treatment: a structured telephone interview study

**DOI:** 10.1186/s12904-020-00591-4

**Published:** 2020-06-19

**Authors:** Adinda Mieras, Bregje D. Onwuteaka-Philipsen, Annemarie Becker-Commissaris, Jose C. M. Bos, H. Roeline W. Pasman

**Affiliations:** 1 Amsterdam UMC, Vrije Universiteit Amsterdam, Department of Pulmonary Diseases, Cancer Center Amsterdam, Amsterdam, The Netherlands; 2 Amsterdam UMC, Vrije Universiteit Amsterdam, Department of Public and Occupational Health, Amsterdam Public Health research Institute, Amsterdam, The Netherlands; 3 Amsterdam UMC, Vrije Universiteit Amsterdam, Expertise Center for Palliative Care, Amsterdam, Netherlands; 4 Public Health research Institute, Expertise Center for Palliative Care, de Boelelaan 1117, 1081 HV Amsterdam, Netherlands; 5 Dijklander Ziekenhuis, Department of Pulmonary Diseases, Purmerend, The Netherlands

**Keywords:** Treatment goals, Lung cancer, Chemotherapy, Targeted therapy, Immunotherapy, End of life, Family

## Abstract

**Background:**

Lung cancer has a high impact on both patients and relatives due to the high disease burden and short life expectancy. Previous studies looked into treatment goals patients have before starting a systemic treatment. However, studies on relatives’ perceptions of treatment at the end of life are scarce. Therefore, we studied the perspectives of relatives in hindsight on the achievement of treatment goals and the choice to start treatment for metastatic lung cancer of their loved one.

**Methods:**

We conducted a structured telephone interview study in six hospitals across the Netherlands, one academic and five non-academic hospitals, between February 2017 and November 2019. We included 118 relatives of deceased patients diagnosed with metastatic lung cancer who started a systemic treatment as part of usual care (chemotherapy, immunotherapy or targeted therapy with tyrosine kinase inhibitors (TKIs) and who completed a questionnaire on their treatment goals before the start of treatment and when treatment was finished. We asked the relatives about the achievement of patients’ treatment goals and relatives’ satisfaction with the choice to start treatment. This study is part of a larger study in which 266 patients with metastatic lung cancer participated who started a systemic treatment and reported their treatment goals before start of the treatment and the achievement of these goals after the treatment.

**Results:**

Relatives reported the goals ‘quality of life’, ‘decrease tumour size’ and ‘life prolongation’ as achieved in 21, 37 and 41% respectively. The majority of the relatives (78%) were satisfied with the choice to start a treatment and even when none of the goals were achieved, 70% of the relatives were satisfied. About 50% of relatives who were satisfied with the patients’ choice mentioned negative aspects of the treatment choice, such as the treatment did not work, there were side effects or it would not have been the relatives’ choice. Whereas, 80% of relatives who were not satisfied mentioned negative aspects of the treatment choice. The most mentioned positive aspects were that they tried everything and that it was the patient’s choice.

**Conclusion:**

The majority of relatives reported patients’ treatment goals as not achieved. However, relatives were predominantly satisfied about the treatment choice. Satisfaction does not provide a full picture of the experience with the treatment decision considering that the majority of relatives mentioned (also) negative aspects of this decision. At the time of making the treatment decision it is important to manage expectations about the chance of success and the possible side effects of the treatment.

## Background

Lung cancer is the world’s leading cause of cancer death [1]. For patients with metastatic lung cancer chemotherapy, immunotherapy and targeted therapy with tyrosine kinase inhibitors (TKIs) are possible palliative systemic treatments with the aim of relieving symptoms, temporary disease control and prolonging survival [2–5].

People at the end of life often have diverse physical, psychological and social needs, as well as a need to prepare for death and achieve peace at the end of life [6–8]. While patients and relatives attach great value to fulfilling these needs [8], at the same time there is often hope for a cure or life prolongation [9] (Mieras et al.: What goals do patients and oncologists have when starting a medical treatment for metastatic lung cancer?, submitted).

Three studies found the following treatment goals that patients mentioned before starting treatment for metastatic lung cancer: improve or maintain quality of life, prolong life, find comfort, fight cancer and cure cancer [10–12]. In a previous study we found that after treatment patients reported in less than 50% of time that these goals were achieved: quality of life for 30%, life prolongation for 49%, decrease tumour size for 26% and cure for 44%. Directly after the treatment was finished most patients felt, in hindsight, that starting treatment was the right decision, even if the treatment goals were not achieved (Mieras et al.: What goals do patients and oncologists have when starting a medical treatment for metastatic lung cancer?, submitted).

Metastatic lung cancer has a large impact on both patients and relatives [13, 14]. Relatives often accompany patients to a physician visit and help the patients obtain information relevant to medical treatments [15–17]. Relatives might have an alternative opinion to the patient regarding the choice to start treatment and whether the goals were actually achieved. The relatives witness the patient with metastasized lung cancer from diagnosis to death, and they are able to take into account the last phase of life when considering whether treatment goals are achieved and if the right choice was made. Additionally, the relative has a different perspective since they are not the patient.

Since metastatic lung cancer also affects the life of patient’s relatives and not much is known on their views in hindsight the objectives were to study the perspective of relatives on the choice to start lung cancer treatment, after the patient had deceased. We specifically focussed on (1) relatives’ perspective regarding achievement of patients’ treatment goals, (2) relatives’ view on the patients’ choice to start treatment and (3) the relation between the achievement of treatment goals and satisfaction with the patient’s choice to start treatment.

## Methods

### Study design and population

The present study is an explorative sub-study of a larger prospective study on achievement of the goals metastatic lung cancer patients and their oncologists have when starting a palliative systemic treatment as part of usual care (chemotherapy, immunotherapy or targeted therapy with tyrosine kinase inhibitors (TKIs)) (Mieras et al.: What goals do patients and oncologists have when starting a medical treatment for metastatic lung cancer?, submitted). During the patients’ informed consent procedure in the previous study, patients and relatives were asked to choose a relative to participate in the present study if the patient is deceased. Inclusion criteria were ability to give informed consent, possessing a telephone and willing to participate. During the structured telephone interview with relatives of deceased metastatic lung cancer patients we asked to what extent they felt that the goals patients had when starting a systemic treatment were achieved. The telephone interview was conducted with relatives a minimum of 6 weeks after the patient had died, which we feel is an appropriate time frame to be able to recall the treatment, but it not too early after a loved one has passed. To enhance the rigor in the study, the researcher who interviewed the relatives was transparent, i.e. not the treating physician of the patient. Additionally, the interviews were all conducted in the same way, following the same order in the questionnaire and performed by one researcher (AM, between February 2017 and November 2019).

### Data collection

The structured telephone interview schedule was developed based on the questionnaire for patients and oncologists (see appendix 1). During the structured telephone interviews, questions were read aloud and answers were written down textually. The interviews were not audio recorded. The interviews focused on the treatment goal(s) the patient reported before the start of treatment. During the interview relatives were asked to what extent they perceived the patients’ treatment goal(s) as achieved on a scale from 0 to 10, with 0 as not achieved at all and 10 as completely achieved. Additionally, relatives were asked if they were satisfied with the patients’ choice to starting treatment and whether the relatives thought they received enough information on the given treatment of the patient (see appendix). The relatives’ age, gender and relation to the patient were documented.

The time between the patient’s death and the interview with their relative was on average 86 days, with the exception of one relative that was interviewed 15 days after the patient died, instead of 6 weeks after the patient died, because the investigator did not know the patient had already died when she called for the study among patients. The relative preferred to do the interview at that time rather than later. The time between the last administration of treatment the patient received and the interview with the relative was on average 201 days.

### Ethics, consent and permission

This study was approved by the medical ethical committee (METc) of the VU University Medical Centre in Amsterdam, the Netherlands (number NL57455.029.16). Both patients and relatives gave written consent to participate in the study and for patients to have their medical records reviewed. Relatives were able to withdraw their consent at any time.

### Data analysis

Descriptive statistics were conducted using IBM SPSS statistics 24. We perceived a goal as achieved if it was rated with a 7 or higher (on a 0 to 10 scale). This was based on the semi-structured interviews performed in the previous study in which we asked the patients to what extent they felt their treatment goals were achieved on a scale from 0 to 10 (Mieras et al.: Patients with metastatic lung cancer and oncologists views on achievement of treatment goals and making the right treatment decision: a prospective multicentre study, submitted). The question on the satisfaction with the treatment choice in hindsight was to be answered with ‘yes’, ‘no’ or ‘I am not sure’ and additionally open-ended for further explanation. The answers ‘no’ and ‘I am not sure’ were for analysis merged into ‘not satisfied’. Answers to this open-ended question were categorized independently and subsequently compared by 4 research members (HRWP, BDO, AM, AB). The codes agreed upon were grouped in the categories ‘positive aspects’, ‘negative aspects’ and ‘other aspects’. Discrepancies were resolved through discussion until 100% agreement was achieved.

## Results

### Participant recruitment

**Fig. 1:**
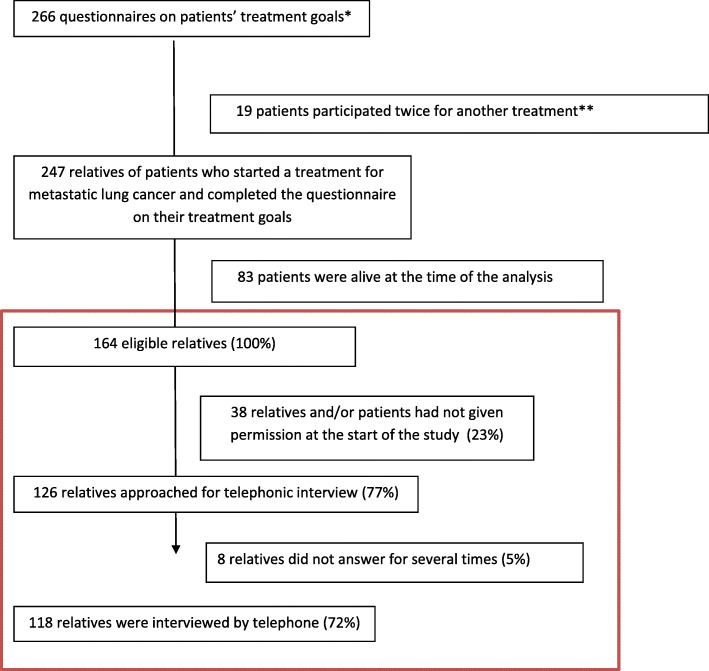
Flowchart of participants. The red box concerns the participants in the current study which is part of a larger study in which 266 patients with metastatic lung cancer participated. In that study, patients could participate multiple times in the study resulting in 247 individual patients from whom the relatives could be approached. Only relatives of deceased patients were eligible to participate. In the end, 118 relatives participated in this interview study. * consent from patient and relative was asked at the start of the treatment. ** Patients were allowed to participate twice in the questionnaire study when they sequentially received another treatment

In total, 266 patients started a treatment for metastatic lung cancer and completed the questionnaire on their treatment goals. Of these patients, 164 patients were deceased during the study period, resulting in 164 relatives being eligible for participation of which 118 (72%) participated in an interview (Fig. 1). Refusing of participation during the informed consent procedure was in most of the cases because the patient didn’t want to bother their relatives with the study in which he or she participated. Decline in participation during the telephonic interview was because the relative already died, telephonic number was not in use or the phone was not picked up after trying several times on different days and times.

### Characteristics of study participants

Participants had an average age of 62 years and ranged between 30 and 85 years. Most participants were female (63%) and the partner of the patient (81%) (Table 1). Length of the interview was not recorded but roughly lasted between 5 and 60 min with an average time of 15 min.

**Table 1:** Demographic characteristics of study participants

Variable	Numeber	Percent
Participants	118	100
Age – Years		
Mean ± SD	62 ± 11	
Range	30–85	
Sex		
Male	43	37
Female	75	63
Relation to patient		
Partner	96	81
Father/mother	1	1
Son/daughter	16	14
Sibling	4	3
Friend	1	1

### Achievement of patients’ treatment goals according to relatives

**Fig. 2:**
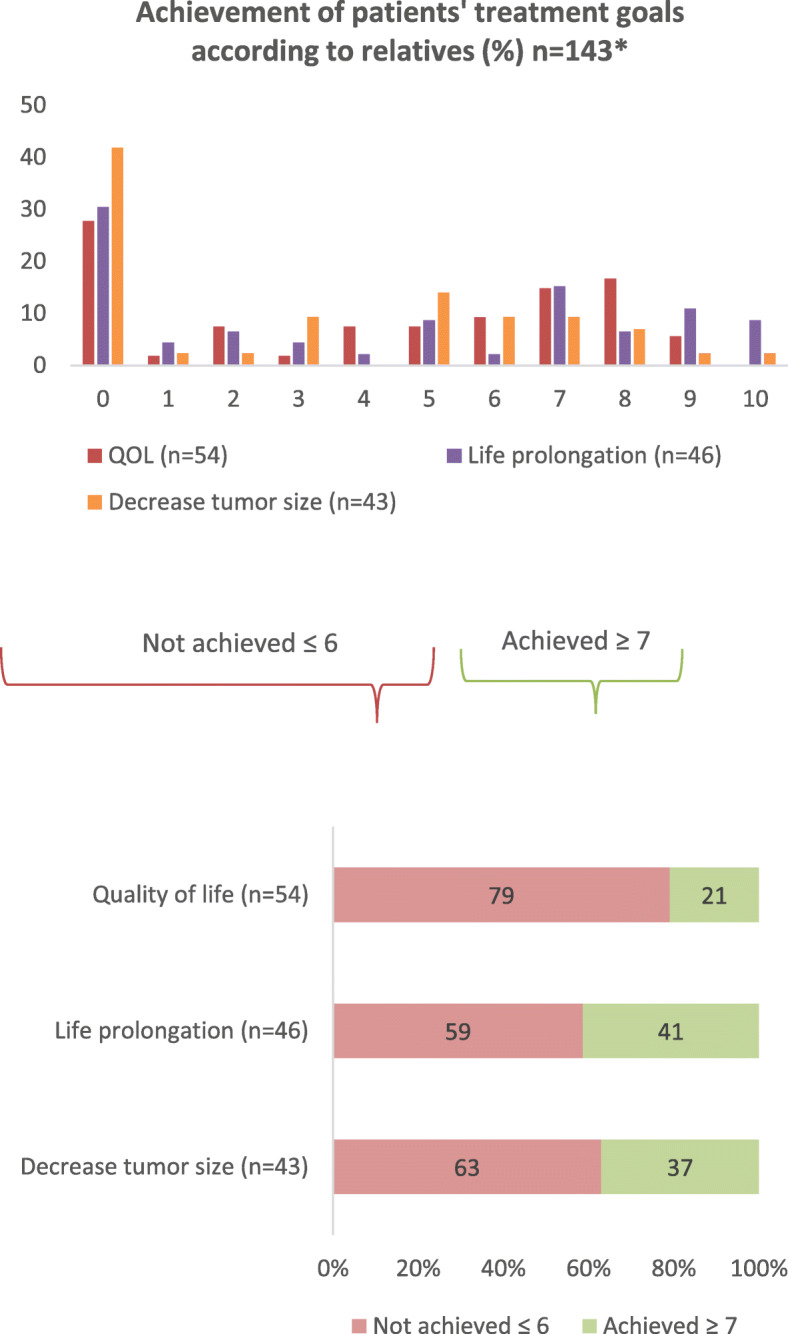
Achievement of the patients’ treatment goals according to relatives on a scale from 0 to 10 with 0 meaning ‘not achieved at all’ and 10 meaning ‘totally achieved’. These number are dichotomized into achieved and not achieved with a cut off score of 7. * Relatives (*n* = 118) had answered on 143 treatment goals together

The 118 relatives of patients reported about the achievement of 143 treatment goals with an average of 1.2 goals per patient and a maximum of three goals. In total, 21 patients mentioned ‘cure’ as a treatment goal. Since only the relatives of deceased patients were included, we did not ask if the goal ‘cure’ was achieved so we excluded this treatment goal from the analysis. Relatives overall most often reported the achievement of the treatment goal with 0 (not achieved at all) (n = 47). Relatives reported the goals ‘quality of life’, ‘life prolongation’ and ‘decrease in tumour size’ as achieved in 21, 41 and 37% respectively (Fig. 2). In total, 76 relatives (64%) perceived none of the goals as achieved, 42 relatives (36%) reported that at least one of patients’ goals was achieved, and 29 relatives (25%) reported that all goals were achieved (data not shown).

### Satisfaction with patients’ choice to start treatment

A total of 78% (n = 92) of the relatives was, in hindsight, satisfied with the patients’ choice to start treatment, 14% (n = 16) was not satisfied about the treatment choice and 9% (n = 10) was not sure. When asked to explain why they felt satisfied or not with the patients’ treatment choice, 42% of relatives reported only positive aspects, 31% reported both positive and negative aspects and 27% reported only negative aspects. Relatives that were satisfied with the treatment choice did not only mentioned positive aspects: 30% mentioned both positive and negative aspects and 22% mentioned only negative aspects. Furthermore, relatives that were not satisfied did not only mentioned negative aspects: 36% mentioned both positive and negative aspects and 20% mentioned only positive aspects.

The most frequently mentioned positive aspects were ‘we tried everything’ (24%), ‘it was the patient’s choice’ (14%), and ‘the patient lived longer’ (14%). ‘It was the patient’s choice’ was more frequently mentioned by relatives that were not satisfied with the treatment choice than relatives that were satisfied (23% versus 12%). ‘The patient lived longer’ was more frequently mentioned by relatives who were satisfied than relatives who were not (16% versus 4%).The most frequently mentioned negative aspects were ‘the treatment did not work’ (19%), ‘there were side effects’ (13%), and ‘it was not my choice’ (10%). Next to positive and negative aspects relatives also mentioned other aspects, which were often related to the quality of care. Insufficient quality of care was more frequently mentioned by relatives who were not satisfied than relatives who were (23% versus 10%). Good quality of care was only mentioned by relatives that were satisfied (21%) (Table 2). Most of the relatives (78%) were satisfied with the choice to start a treatment (n = 92). From the relatives who reported that at least one of patients’ goals was achieved (n = 42), 93% were satisfied about the treatment choice. When none of the goals were achieved (n = 76), 70% of the relatives were satisfied.

**Table 2:** Explanations of being satisfied with the treatment choice

N column (%)	Total (n = 118) N (%)	Satisfied (n = 92) N (%)	Not satisfied^a^ (n = 26) N (%)
Relatives mentioned: ^b^			
• Only positive aspects	47 (42)	42 (48)	5 (20)
• Positive and negative aspects	35 (31)	26 (30)	9 (36)
• Only negative aspects	31 (27)	19 (22)	11 (44)
Positive aspects mentioned			
• We tried everything	28 (24)	22 (24)	6 (23)
• It was the patients’ choice	17 (14)	11 (12)	6 (23)
• Lived longer	16 (14)	15 (16)	1 (4)
• The treatment worked	14 (12)	11 (12)	3 (12)
• No side effects	12 (10)	10 (11)	2 (8)
• It gives hope	10 (8)	7 (8)	3 (12)
• Good quality of life	6 (5)	6 (7)	0 (0)
• Other	3 (3)	3 (3)	0 (0)
Negative aspects mentioned			
• The treatment didn’t work	23 (19)	17 (18)	6 (23)
• Side effects	15 (13)	11 (12)	4 (15)
• It was not my choice	12 (10)	5 (5)	7 (27)
• Quality of life worsened	10 (8)	3 (3)	7 (27)
• Treatment was given too long	9 (8)	4 (4)	5 (19)
• Burdensome hospital visits	5 (4)	3 (3)	2 (8)
• The treatment was started too late	4 (3)	4 (4)	0 (0)
Other aspects mentioned			
• Good quality of care	19 (16)	19 (21)	0 (0)
• Insufficient quality of care	15 (13)	9 (10)	6 (23)
• It was the choice of the oncologists	13 (11)	11 (12)	2 (8)
• I don’t know how it would have been otherwise	7 (6)	3 (3)	4 (15)
• It helped science	3 (3)	3 (3)	0 (0)

^a^Including relatives who reported “not sure” on the treatment satisfaction. ^b^ 4% missing. Percentages don’t add up to 100% since more answers were possible

***Examples of explanation of relatives who answered ‘satisfied about the treatment choice’ and mentioned:***

- ***Only positive aspects:***
  *You try to get hope with the treatment. He had no side effects, as with chemotherapy. However, he progressed after a couple of cycles. We started the treatment to stall/extend. Every 3 weeks. Eventually he passed away through euthanasia, he wanted to keep control in his own hands (****in the categories ‘It gives hope’ and ‘no side effects; relative of deceased patient, age between 50 and 60 years****)*
- ***Positive and negative aspects****:*
  *It was my husbands’ choice, so that is good. But for me, it all went really fast. Doctors repeatedly said: it is going fine. Then, after surgery on his chest wound it suddenly went wrong and he quickly past away. It is really unfortunate/a shame, and I was angry, because we had not spoken to the oncologists when my husband was admitted to the hospital. No call, no visit. We had to hear from the ward doctor that there were no treatment options left and he would have very short time left (to live). But, he still had a good summer after the immunotherapy so that is nice (****in the categories ‘It was the patients’ choice’, ‘good quality of life’ and ‘insufficient quality of care’; relative of deceased patient, age between 60 and 70 years****).*
- ***Only negative aspects:***
  *In the final period we had our doubts. The exams took long and in the meantime the cancer kept growing in the liver. After 3 months of radiation of the lungs nothing happened. In the medical files it said palliative chemotherapy, however, this was not mentioned to us (curative). Chemotherapy is still junk, in hindsight the last chemotherapy was too much (****in the categories ‘The treatment was started too late’ ‘insufficient quality of care’ and ‘treatment was given too long’; relative of deceased patient, age between 40 and 50 years****).*

***Examples of relatives who answered ‘not satisfied about the treatment choice’ and mentioned:***

- ***Only positive aspects:***
  *It was his choice. He lived longer because of the chemo. His wife wanted to try. But at a certain point he did not want to anymore, also no immunotherapy unless it was possible from home (****in the categories ‘Lived longer’ and ‘choice of the patient’; relative of deceased patient, age between 50 and 60 years****).*
- ***Positive and negative aspects:***
  *My husband was so ill and he already received so many treatments. There were no more treatment options left, he felt like a test subject, it is tough, traveling long distance and we kept going/continued too long. In [other hospital] they also continue treatment for a long time. But everyone tries to grab on every straw/chance (****in the categories ‘We tried everything’, ‘quality of life worsened’, ‘insufficient quality of care’ and ‘burdensome hospital visits’; relative of deceased patient, age between 70 and 80 years****).*
- ***Only negative aspects:***
  *It didn’t work. She still had lots of treatments after this one. From the chemo she only lost her hair/ turned balled (****in the categories ‘The treatment didn’t work’ and ‘side effects’; relative of deceased patient, age between 60 and 70 years****).*

## Discussion

Relatives reported the goals ‘quality of life’, ‘decrease tumour size’ and ‘life prolongation’ as achieved in 21, 37 and 41% respectively. Most of the relatives (78%) were satisfied about the patients’ choice to start treatment. Even if none of the goals were achieved, 70% of the relatives were satisfied. In total, 52% of relatives who were satisfied with the patient’s choice mentioned negative aspects of the treatment choice, such as that the treatment did not work, that there were side effects or that it would not have been the relatives choice. While 80% of relatives who were not satisfied with the patient’s choice to start treatment mentioned negative aspects. The most mentioned positive aspects were that they tried everything and that it was the patient’s choice. In total, 31% of relatives reported both positive and negative aspects of the treatment choice, independently of being satisfied or not.

### Relatives consider patient’s treatment goals less often achieved than patients

The patients for whom the relatives reported whether the treatment goals were achieved reported their predefined treatment goals ‘quality of life’, ‘decrease tumour size’, and ‘life prolongation’ achieved in 30, 26 and 49% respectively (Mieras et al.: What goals do patients and oncologists have when starting a medical treatment for metastatic lung cancer?, submitted). Thus, compared to patients, relatives consider the goals ‘quality of life’ (21% vs 30%) and ‘life prolongation’ (41% vs 49%) less often achieved and ‘decrease tumour size’ (37% vs 26%) more often achieved. This difference could firstly be due to the fact that relatives reported the achievement of goals after the patient died taking into account the whole illness. For the goal ‘quality of life’, for example, it might be that at the time the treatment stopped (the time point that the patient reported the achievement of goals) the quality of life of the patient was higher compared to the last phase of life. The latter was most likely the reference point for relatives when they considered whether the goal ‘quality of life’ was achieved. Secondly, relatives may have a different perspective on quality of life because they have their own believes and considerations to undergo or forgo cancer treatment than patients. Thirdly, they do not have the disease themselves, which may also influence their perspective. Fourthly, when looking at the negative aspects, relatives mentioned side effects and worsened quality of life It might also be that from the perspective of relatives it is very difficult seeing their loved one deteriorate and suffer and therefore more often report the goal ‘quality of life’ not achieved. Finally, it is known that relatives tend to assess a patient’s quality of life as somewhat lower than what the patient perceives [18, 19].

### Satisfaction with treatment decision is linked to negative feelings about treatment decision

Most of the relatives were, in hindsight, satisfied about the patients’ choice to start treatment (78%), even if none of the goals were achieved (70%). These results are comparable to the patients’ and oncologists’ view regarding making the right decision to start treatment (patients: 79% and oncologist: 96%) even if none of the goals were achieved (patients: 72% and oncologists: 93%) in the previous study of Mieras et al (Mieras et al.: What goals do patients and oncologists have when starting a medical treatment for metastatic lung cancer?, submitted). It is known that measuring satisfaction is not without problems. It holds the risk of creating a positive bias which could, for instance, be influenced by the desire to give a socially desirable answers or, according to cognitive dissonance theory, a tendency to assess one’s situation or actions as good in hindsight [20, 21]. Nevertheless, the fact that we found over half of people who were satisfied with the treatment decision described negative aspects related to the decision taken shows that satisfaction does not encompass the relatives’ entire experience. The most mentioned negative aspects were that the treatment did not work and that there were burdensome side effects of the treatment. These negative aspects should be taken into account when deciding to start a treatment with a relatively low chance of success and high chance of side effects e.g. by managing expectations of patients and relatives with clear communication and highlighting the option of palliative or supportive care to treat side effects.

Next to negative aspects related to the treatment decision taken, many relatives also mentioned positive aspects. It was most frequently mentioned that it was positive the patient tried everything. Previously, we found that this was also an important aspect for the patients and oncologists (Mieras et al.: What goals do patients and oncologists have when starting a medical treatment for metastatic lung cancer?, submitted). Additionally, it was important for many relatives that the patients’ wish for treatment was followed, even when the relatives themselves felt that the treatment might have gone on for too long. Notably, none of the positive aspects we found resonated with aspects valued at the end of life found in a study by Steinhauser et al.: ‘pain and symptom management’, ‘clear decision making’, ‘preparation for death’ and ‘completion’ valued at the end of life [8]. This might be related to the fact that in our study we focused on the evaluation of the decision to start systemic treatment. For patients who start with treatment and their families, it might be more difficult to prepare for death than for people who do not start treatment. It might also be that they value the aspects at the end of life less.

### Strengths and limitations

A strengths of this study is that it provides new insights in the perspective of the relative on the treatment for an incurable disease of their loved one; we could not find studies which results we could compare to ours. Furthermore the contribution of both one academic and five non-academic hospitals (multi-centre and a case mix of patients) and the adequate number of participants at a difficult time willing to share information on a sensitive subject contribute to the validity of the results. Another strength is that through the structured telephone interviews with open questions, the relatives were allowed to elucidate positive and negative aspects on the treatment satisfaction. Since all interviews were conducted by one researcher continuity is assured, however, it may also cause interpretation bias which can be seen as a limitation. Another limitation might be that the researcher wrote down the answers during the telephone interview and might not have managed to capture all the details, instead of when the conversations were audio recorded.

## Conclusions

The majority of relatives reported patients’ treatment goals as not achieved. However, relatives were predominantly satisfied about the treatment choice. Satisfaction of treatment choice does not encompass the entire experience with the treatment decision since the majority of relatives mentioned negative aspects of this decision. At the time of making the treatment decision it is important to manage expectations about chance of success and possible side effects of the treatment. Relatives, like patients, find it important to feel that something is being done, thus, it can be beneficial to not contrast the option of systemic treatment with the option of doing nothing. Palliative care can also be framed as a treatment option.

## Supplementary information

**Additional file 1.** Appendix. Questionnaire for relatives.

## Data Availability

The datasets used and/or analysed during the current study are available from the corresponding author on reasonable request.

## Abbreviations

TKI: Tyrosine kinase inhibitors
METC: Medical ethical committee
KWF: Dutch Cancer Society
ZonMw: The Netherlands Organisation for Health Research and Development
