# Supplementary material for: Relatives of deceased patients with metastatic lung cancer’s views on the achievement of treatment goals and the choice to start treatment: a structured telephone interview study
Source: BMC Palliat Care. 2020 Jun 19;19:86. doi: 10.1186/s12904-020-00591-4 (PMC7305592; doi:10.1186/s12904-020-00591-4)
Supplement: Supplementary file 1 — Additional file 1. Appendix. Questionnaire for relatives. [file 12904_2020_591_MOESM1_ESM.docx]

**Appendix**

**Questionnaire for relatives:**

- *“In hindsight, are you satisfied with the treatment choice of your relative? Can you tell me why you are or are not satisfied?”*

Yes, because:

No, because:

I am not sure, because:

- “*In hindsight, what do you think of the information you received on your relatives treatment? Did you receive enough information or what information did you miss*?”

Too much:

Enough:

Not enough, I missed:

- “*Your relative reported the following goal(s) before he or she started treatment. To what extent do you think the goal is achieved by/through the treatment? It is on a scale from 0 to 10 with 0 meaning not achieved at all and 10 meaning entirely achieved*”

“*Read goal 1 aloud*”: 0 1 2 3 4 5 6 7 8 9 10

[Room for explanation]

“*Read goal 2 aloud*”: 0 1 2 3 4 5 6 7 8 9 10

[Room for explanation]

“*Read goal 3 aloud*”: 0 1 2 3 4 5 6 7 8 9 10

[Room for explanation]

“*Read goal 4 aloud*”: 0 1 2 3 4 5 6 7 8 9 10

[Room for explanation]
